# Comparison of annual percentage change in breast cancer incidence rate between Taiwan and the United States—A smoothed Lexis diagram approach

**DOI:** 10.1002/cam4.1102

**Published:** 2017-05-31

**Authors:** Li‐Hsin Chien, Tzu‐Jui Tseng, Chung‐Hsing Chen, Hsin‐Fang Jiang, Fang‐Yu Tsai, Tsang‐Wu Liu, Chao A. Hsiung, I‐Shou Chang

**Affiliations:** ^1^ Division of Biostatistics and Bioinformatics Institute of Population Health Sciences National Health Research Institutes Taiwan; ^2^ Center of Biomedical Resources National Health Research Institutes Taiwan; ^3^ National Institute of Cancer Research National Health Research Institutes Taiwan

**Keywords:** Age effects, annual percentage change in rates, breast cancer, cohort effects, period effects

## Abstract

Recent studies compared the age effects and birth cohort effects on female invasive breast cancer (FIBC) incidence in Asian populations with those in the US white population. They were based on age–period–cohort model extrapolation and estimated annual percentage change (EAPC) in the age‐standardized incidence rates (ASR). It is of interest to examine these results based on cohort‐specific annual percentage change in rate (APCR) by age and without age–period–cohort model extrapolation. FIBC data (1991–2010) were obtained from the Taiwan Cancer Registry and the U.S. SEER 9 registries. APCR based on smoothed Lexis diagrams were constructed to study the age, period, and cohort effects on FIBC incidence. The patterns of age‐specific rates by birth cohort are similar between Taiwan and the US. Given any age‐at‐diagnosis group, cohort‐specific rates increased overtime in Taiwan but not in the US; cohort‐specific APCR by age decreased with birth year in both Taiwan and the US but was always positive and large in Taiwan. Given a diagnosis year, APCR decreased as birth year increased in Taiwan but not in the US. In Taiwan, the proportion of APCR attributable to cohort effect was substantial and that due to case ascertainment was becoming smaller. Although our study shows that incidence rates of FIBC have increased rapidly in Taiwan, thereby confirming previous results, the rate of increase over time is slowing. Continued monitoring of APCR and further investigation of the cause of the APCR decrease in Taiwan are warranted.

## Introduction

Breast cancer incidence rate is rapidly increasing. It is the most frequently diagnosed cancer in Asian countries and the second leading cause of cancer death among Asian women 1, 2. Previous studies indicated that female invasive breast cancer (FIBC) in Asian women was characterized by an early age at onset and its age‐specific incidence rate had a peak before age 50. This is in contrast to FIBC in Western countries, where the age‐specific incidence rate increases continuously with age 1, 2, 3, 4, 5, 6. Explanations for these phenomena include calendar‐period effects or birth cohort effects 7, 8, 9, 10, and age‐specific etiology 3, 11, 12, 13, 14. In fact, some have proposed that FIBC in Asia might be a disease etiologically different from that in Western countries 5, 12, 15.

Sung et al. (2015) reported that the longitudinal age‐specific incidence rates of FIBC in certain Asian populations appear to be proportional to those in the United States, and the incidence rates in recent cohorts in Asian countries are converging or even surpassing the historically high US rates 16. The authors noted that the major limitation of their study was that the longitudinal age‐specific rates were extrapolated using age–period–cohort models 16, 17. They also noted the needs to quantify the proportion of incidence rate increases in Asian countries that are attributable to period and screening effects versus birth cohort effects.

Sung et al. (2016) studied birth cohort effects on FIBC among younger (30–49 years) and older (50–79 years) Chinese populations and US non‐Hispanic white women. They reported that cohort‐specific rates increased in every Chinese population, and that incidence rate rose more rapidly among older than younger women. Their results are based on age–period–cohort model and estimated annual percentage change (EAPC) in the age‐standardized incidence rates (ASR).

Recently, we reported that age‐specific FIBC incidence rates in Taiwan increased monotonically with age in the period 1988–2007 for every birth cohort younger than 1928; for the older birth cohorts, the rates increased initially and sometimes reached a peak before decreasing; if occurring, the peak was around 80 years of age (see Fig. 3A in Ref. 18). Compared with standard approaches, our method based on a smoothed Lexis diagram is more revealing, requires minimum model assumption, and no extrapolation, and performs better in terms of estimation error 18.

The main objectives of this study were to use the smoothed Lexis diagram approach to compare the age, cohort, and period effects on the incidence of FIBC in Taiwan and those in the United States without imposing model assumptions. In particular, we examined age‐specific incidence by cohort, cohort‐specific incidence by age at diagnosis, cohort‐specific annual percentage change in rates (APCR) by age at diagnosis, period‐specific APCR by age at diagnosis, and age‐specific rates by year of diagnosis.

Average annual percentage change in disease rates is useful in the comparison of changes in disease rates and is widely used in cancer surveillance 19, 20, 21. Tarone and Chu used age‐specific biannual percentage change in rates to test the null hypothesis that there are no cohort effects on breast cancer mortality 22. Our approach goes further by directly measuring the effect size of age‐ and cohort‐specific APCR and providing graphical presentations, which is the strength of the smoothed Lexis diagram 23, 24, 25. It was hoped that we could gain more insights into the proportion of the incidence rate increases that are attributable to period effects versus cohort effects.

## Methods

### Study population

In this study, we considered women diagnosed with invasive breast cancer (ICD‐9‐CM‐codes:174.0‐174.9, excluding morphology codes 9050‐9055,9140,9590‐9992). We obtained FIBC case data from the Taiwan Cancer Registry (TCR) and population data from the Taiwan's Ministry of the Interior (accessed January 2015). The TCR was launched in 1979 to collect information for all newly diagnosed cancer cases from hospitals with 50 or more beds. Quality indicators such as the percentage of morphologically verified cases (MV%) and the percentage of death‐certificate‐only cases (DCO%) have all shown a steady improvement of quality in the TCR. For example, the DCO% decreased from 18.5% in 1990–1994, 10.4% in 1995–1999, to 2.8% in 2000–2006. The completeness of the TCR increased from 92.8% in 2002 to 97.7% in 2011 26, 27.

We also obtained FIBC incidence data for non‐Hispanic white women in the United States from SEER 9 Research Data. The SEER 9 registries include Atlanta, Connecticut, Detroit, Hawaii, Iowa, New Mexico, San Francisco‐Oakland, Seattle‐Puget Sound, and Utah. All SEER registries are obliged to meet the Gold Standard Registry Certification from the North American Association of Central Cancer Registries, Inc., for completeness, accuracy, and timeliness of data 28. The SEER 9 registries represent approximately 10% of the US population. The case ascertainment is estimated to be 98% 29.

For a more reliable comparison, we followed the same criteria in selecting study subjects from the TCR and SEER 9, namely, cases diagnosed between 1991 and 2010 and with an age at diagnosis between 30 and 84. In total, there were 98,489 newly diagnosed patients in the TCR and 287,472 in SEER 9. Based on the TCR and Taiwan population data, we constructed, respectively, the 1‐year tabulated incidence table, providing the number of newly diagnosed FIBC cases for each calendar year and each age group and the 1‐year tabulated demography table, providing the number of women not having been diagnosed with FIBC for each age group in each calendar year. Taiwan FIBC table was done onsite in the Data Science Center, Ministry of Health and Welfare, Taiwan. We constructed similar incidence and demography tables for the United States based on SEER 9. This study was approved by the institutional review board of the National Health Research Institutes, Taiwan.

### Smoothed Lexis diagram

Given a population and a specific cancer, the standard Lexis diagram reports the incidence rate of a disease in terms of the number of new cases per 100,000 person‐years for each age group and each group of calendar year of diagnosis. To make the data less volatile, Lexis diagrams with 5‐year intervals for age group and 5‐year intervals for calendar year of diagnosis are often considered. One important use of standard Lexis diagrams is to describe the effects of age, period, and cohort on incidence rates in terms of the classical graphical displays. The most frequently reported graphic displays include (1) age‐specific rates by year of diagnosis (rates vs. age, observations within each period connected, i.e., cross‐sectional age‐specific rates); (2) age‐specific rates by year of birth (rates vs. age, observations within each birth cohort connected, i.e., longitudinal age‐specific rates); (3) year‐specific rates by age of diagnosis (rates vs. period, observations within each age‐class connected); (4) cohort‐specific rates by age of diagnosis (rates vs. cohort, observations within each age‐class connected) 25.

The usefulness of these plots are well‐known. For example, because different birth cohort may reflect different risk factor exposure, the effect of age on disease incidence can be best studied by considering the incidence for each birth cohort separately, motivating the study of age‐specific rates by year of birth. Since age is an important risk factor for cancer, comparing incidence rate for people of the same age but from different birth cohort is a useful way to assess changes in disease burden. It is well‐known that these graphic displays based on standard Lexis diagram, involving a simple smoothing approach, suffer from a significant limitation; important details may be lost in the averaging process involved in generating a summary rate, and these details may be useful in understanding time trends in disease 23.

To overcome this drawback, we proposed smoothed Lexis diagram, which is a smooth function *F*(*x*,* y*) that reports the probability that an individual will be newly diagnosed with this cancer at age *x* in calendar year *y* in this population. It was shown by simulation studies that smoothed Lexis diagram performs better than standard ones in terms of estimation error 18. With proper transformation, both *x* and *y* are equal‐spaced fraction numbers in [0,1]. In this paper, patients were diagnosed between 1991 and 2010 and aged between 30 and 84; *x* takes the 20 possible values 0, 1/19, …, 1 and *y* takes the 55 possible values 0, 1/54, …, 1. Although we considered mainly these 1010 lattice points, it is conceptually advantageous to consider (*x*,* y*) any points in the unit square [0,1]*x*[0,1].

Once a smoothed Lexis diagram is obtained, we follow the tradition to present graphically the effects of age, period, and cohort on incidence rates and their 95% credible set. Since these graphs are smooth, they are more revealing in the recognition of patterns. Readers interested in the detail of the construction of the incidence function can referr to the Data S1 or our earlier report 18.

### APCR of incidence rates

To further take advantage of smoothed Lexis diagram in this study, we introduce the APCR for people diagnosed with the disease at aged *x* in period *y*, denoted by *APCR*(*x*,* y*) and defined as

$$\frac{F(x,y+1/19)-F(x,y)}{F(x,y)}.$$

Namely, *APCR*(*x*,* y*) reports, for each age group, the relative increment of incidence from one calendar year to the next. Based on the APCR matrix, we can consider their “classical” plots, such as the cohort‐specific APCR by age *x*, which is the mapping from *c* to *APCR*(*x*,* c* + *x*). Here, *c* denotes a birth cohort.

### Detecting cohort effects by APCR

By its definition, *APCR*(*x,y*) describes a joint effect of the calendar‐period (changes in case ascertainment and/or screening for all age groups at the year *y*) and effect of the birth cohort (changes in risk factors between birth cohorts *y* − *x* and *y* + 1 − *x* during their life up to age *x*). The set {*APCR*(0*,y*), *APCR*(1/55,*y*),…*, APCR*(1,*y*)} was used to explore the relative contribution of period and cohort effects. Roughly speaking, larger variance of this set suggests a larger cohort effect, because period effect does not change with age. If both APCR and cohort effect are positive, then period effect is less than the minimum of the above set. To avoid possible instability of data near boundary, we study period and cohort effects for age between 34 and 80.

### Relationship between cohort‐specific rates and APCRs

Suppose that the cohort‐specific rate by age is unimodal for certain age with its highest rate happening at the birth cohort B. Then differential calculus implies that the cohort‐specific APCR by this age has a value of 0 at the birth cohort B and is decreasing before the birth cohort B. Thus, it is beneficial to monitor whether any cohort‐specific APCR by age is decreasing for early detection of the trend. Only when the cohort‐specific APCR by any age at diagnosis starts to decrease can we expect a future decrease in incidence rates. This point is illustrated in the Figures 2 and 3 on US incidence.

## Results

### Age‐specific rates by cohort

**Figure 1:**
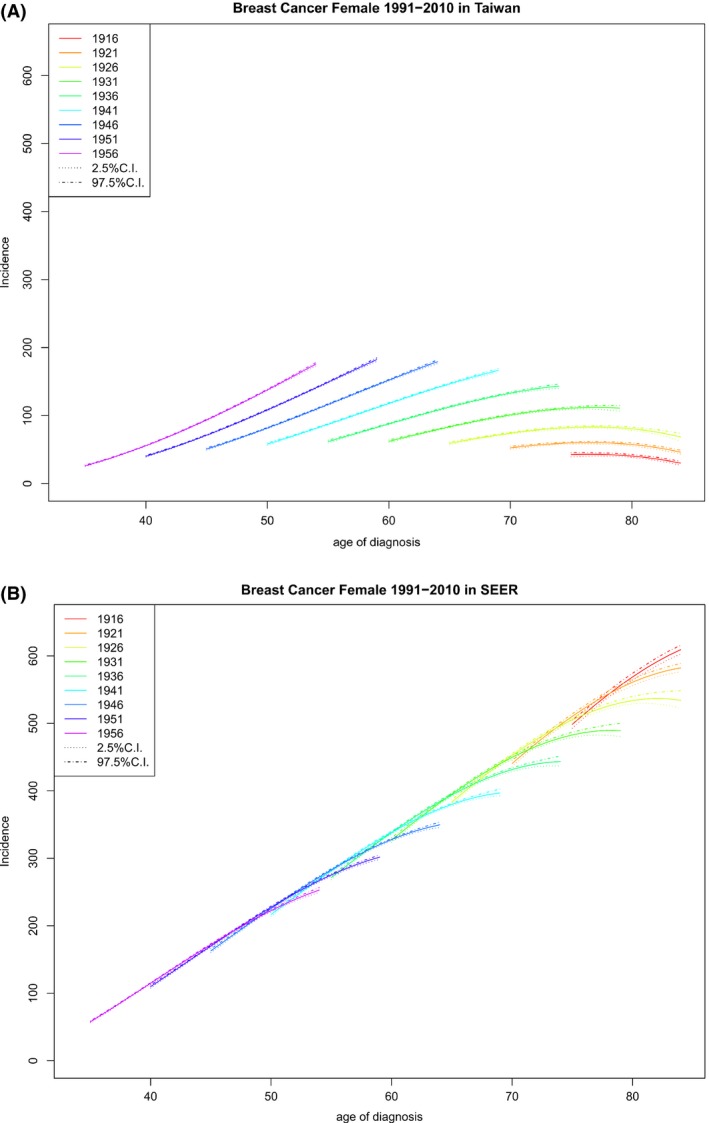
Age‐specific rates by year of birth (rates vs. age of diagnosis, observations within each birth cohort are connected) and their 95% credible intervals. (A) Age‐specific rates by year of birth for Taiwan. (B) Age‐specific rates by year of birth for US SEER‐9.

Figure 1 reports age‐specific rates by birth year, which describes how incidence rate changes with age for each birth cohort and can be used to compare rates among different birth cohorts and different geographic regions.

It follows from Figure 1 that, for any given birth cohort in Taiwan or the United States, the age‐specific rates either increased, initially increased and then decreased, or decreased as age increased; if the age‐specific rates peaked during our study interval, the peak was at an age of 75 years or above.

Figure 1 also shows that for each birth cohort, FIBC incidence rates in Taiwan were lower than those in the United States, but the differences decreased as the birth year increased with the youngest women having the smallest difference, as was also reported previously 16. Table 1 lists the cohort‐specific incidence rate increments in the period 1991–2010 and shows that the differences between these increments in Taiwan and those in the United States decreased as the birth year increased.

**Table 1:** Cohort‐specific incidence rate increment of female invasive breast cancer in the period 1991–2010 in Taiwan and the United States

Cohort	Taiwan		United States	
	Increment	95% CI	Increment	95% CI
1916	−12.67	(−17.50, −8.22)	110.79	(100.68, 120.10)
1921	−6.35	(−10.78, −2.40)	141.76	(133.38, 149.15)
1926	9.50	(4.02, 15.17)	150.88	(135.77, 163.94)
1931	48.82	(44.13, 53.17)	162.21	(150.54, 172.35)
1936	81.76	(77.95, 85.25)	171.97	(163.40, 179.17)
1941	108.32	(105.05, 111.39)	180.16	(173.95, 185.22)
1946	128.50	(125.54, 131.09)	186.78	(182.31, 190.71)
1951	142.30	(139.67, 144.69)	191.83	(188.67, 195.40)
1956	149.71	(147.13, 152.06)	195.31	(191.85, 199.07)
1961	150.74	(147.75, 153.43)	197.22	(193.62, 201.26)

In Taiwan, for any age at diagnosis, recent birth cohorts always had higher rates than earlier cohorts (Fig. 1A); the difference was large, suggesting that there were either cohort effects, period effects, or both. In contrast, in the United States, the difference in age‐specific rates between cohorts was small or negligible for people diagnosed at a younger age and was large only for older people in earlier cohorts; when the difference was not negligible, it was the later birth cohorts that had lower incidence rates (Fig. 1B).

### Cohort‐specific incidence rate by age

**Figure 2:**
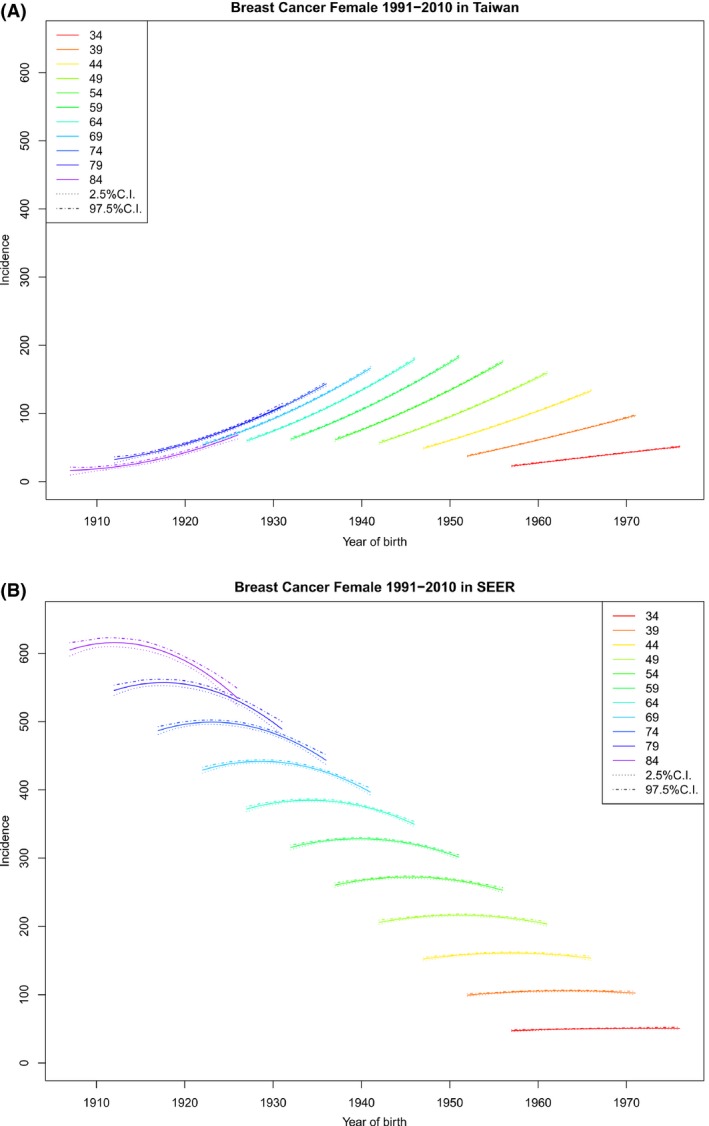
Cohort‐specific rates by age at diagnosis (rates vs. birth cohort, observation within each age group are connected) and their 95% credible intervals. (A) Cohort‐specific rates by age at diagnosis for Taiwan. (B) Cohort‐specific rates by age at diagnosis for US SEER‐9.

Given an age at diagnosis, for the Taiwanese population, the incidence rates increased as the birth year increased (Fig. 2A), whereas US incidence rates first increased and then decreased as the birth year increased if the age at diagnosis was older (Fig. 2B), with the largest decrease appearing among the patients with the oldest age at diagnosis. These patterns can be seen more clearly in Table 2, which reports the age‐specific total rate increment in the period 1991–2010 for Taiwan and the United States. It is evident that in Taiwan the largest increment appeared for patients diagnosed around 64 years of age. This variation in total rate increment between different age groups might be associated with the free breast cancer screening for women aged 45–69 since 2000 in Taiwan (Taiwan Health Promotion Administration, Ministry of Health and Welfare; http://www.hpa.gov.tw/bhpnet/English/ClassShow.aspx?No=201312110001).

**Table 2:** Age‐specific incidence rate increment of female invasive breast cancer in the period 1991–2010 in Taiwan and the United States

Age	Taiwan		United States	
	Increment	95% CI	Increment	95% CI
34	28.36	(26.94, 29.77)	3.41	(1.80, 5.09)
44	84.20	(82.26, 86.13)	1.13	(−2.17, 4.69)
54	114.51	(111.58, 117.26)	−7.44	(−11.09, −3.63)
64	119.29	(116.20, 122.07)	−22.29	(−27.87, −18.14)
74	98.55	(94.64, 102.27)	−43.43	(−52.34, −36.64)
84	52.27	(45.80, 58.90)	−70.84	(−84.70, −57.20)

### Cohort‐specific APCR by age

**Figure 3:**
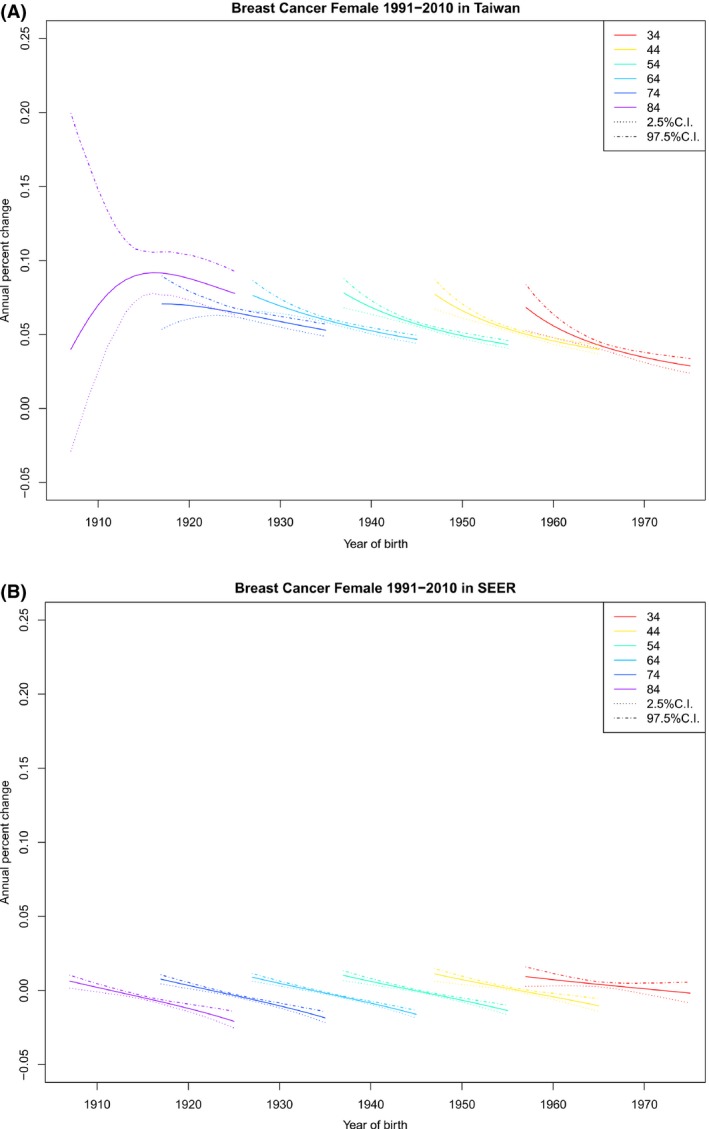
Cohort‐specific APCR by age at diagnosis (APCR vs. year of birth, observations with the same age at diagnosis are connected) and their 95% credible intervals. (A) Cohort‐specific APCR by age at diagnosis for Taiwan. (B) Cohort‐specific APCR by age at diagnosis for US SEER‐9.

Figure 3 depicts cohort‐specific APCR by age for Taiwan and the United States. Although the APCR monotonically decreased as birth year increased for almost every specified age‐at‐diagnosis group in both Taiwan and the United States, the APCR in Taiwan remained positive and large, in contrast to that for the United States, where the APCR was small and became negative in later years. This trend is consistent with the observation that in the United States, birth cohort‐specific incidence rates peaked and started to decrease. In Taiwan, birth cohort‐specific incidence rates monotonically increased with birth year for each age‐at‐diagnosis group (Fig. 2A), whereas the APCR for any age‐at‐diagnosis group decreased with birth year. Thus, in the future, the APCR may become negative, with its incidence rates reaching a peak and then starting to decrease.

### Period‐specific APCR by age and cohort effects

**Figure 4:**
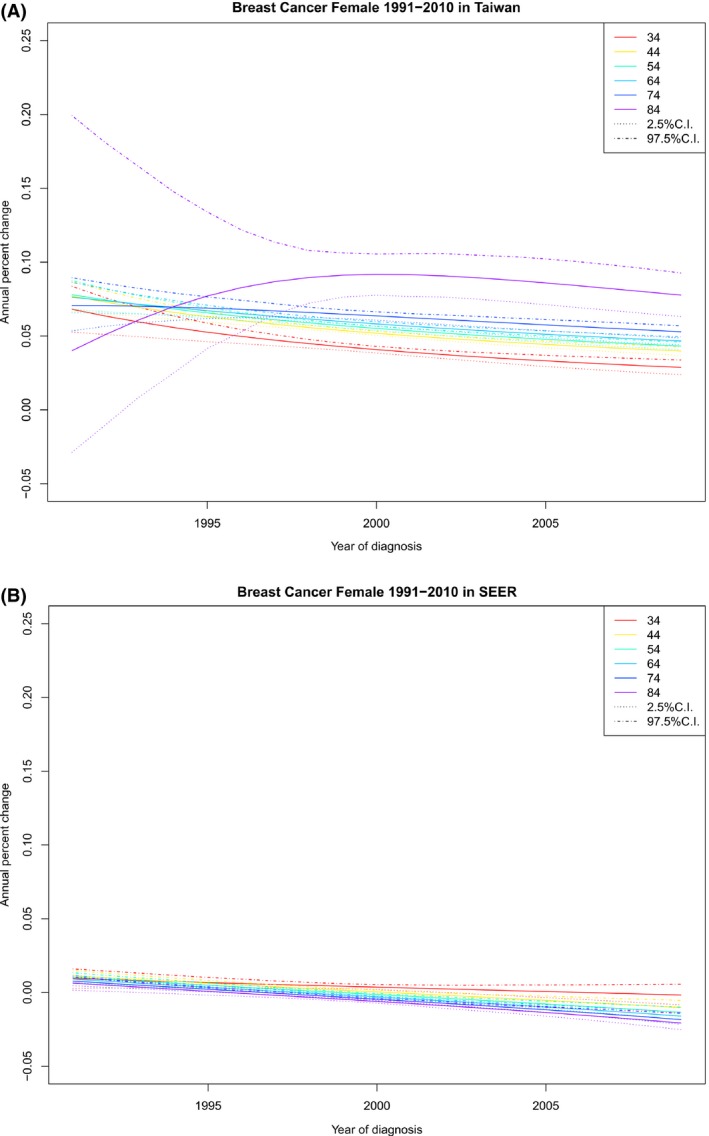
Period‐specific APCR by age at diagnosis (APCR vs. year of diagnosis, observations with the same age at diagnosis are connected) and their 95% credible intervals. (A) Period‐specific APCR by age at diagnosis for Taiwan. (B) Period‐specific APCR by age at diagnosis for US SEER‐9.

Fixing a calendar‐period *y* larger than 1995, we find from Figure 4A that the APCR increased considerably with age in Taiwan. Thus, the joint cohort and period effects at any age was larger than that at age 34, *APCR* (34,*y*). Since it is reasonable to assume that both period and cohort effects were non‐negative 10, we know that period effect was bounded above by *APCR* (34,*y*) for each year. Figure 4B shows that in the United States, the joint period and cohort effects were small, compared with those in Taiwan, and became negative in recent years. The above observation suggests the following assessment of the proportion of APCR that can be attributed to cohort effect.

For each year of diagnosis, the minimum (MIN), the maximum (MAX), the sample mean (SM), and the sample standard deviation (SSD) of the set of the posterior means of the APCR for each cohort in Taiwan are presented in a row of Table 3. Given a row in Table 3, the age that MIN happened, the age that MAX happened, the ratio of MIN to MAX (RATIO‐1), the ratio of MIN to SM (RATIO‐2) are also included in the same row. SM and SSD are presented for the United States. No ratio was reported for the United States, because they may be negative.

**Table 3:** Proportion of incidence rate increase contributable to period effects in Taiwan

Year	Taiwan						United States	
	MIM(age of MIM)	MAX(age of MAX)	RATIO‐1	SM	RATIO‐2	SSD	SM	SSD
(a) Only case ascertainment is considered period effect; APCR for age between 34 and 80.								
1991	0.0603 (80)	0.078 (52)	0.7736	0.0743	0.8115	0.0043	0.0096	0.0014
1992	0.0635 (34)	0.0744 (56)	0.8527	0.0718	0.8836	0.0030	0.0082	0.0015
1993	0.0594 (34)	0.0715 (61)	0.8304	0.0695	0.8547	0.0027	0.0069	0.0016
1994	0.0558 (34)	0.0697 (77)	0.8008	0.0673	0.8291	0.0033	0.0056	0.0017
1995	0.0526 (34)	0.0713 (80)	0.7372	0.0652	0.8065	0.0040	0.0044	0.0018
1996	0.0497 (34)	0.0724 (80)	0.6866	0.0632	0.7863	0.0048	0.0031	0.0019
1997	0.0472 (34)	0.073 (80)	0.6459	0.0614	0.7683	0.0054	0.0019	0.0020
1998	0.0448 (34)	0.0732 (80)	0.6128	0.0596	0.752	0.0060	0.0007	0.0022
1999	0.0427 (34)	0.073 (80)	0.5856	0.0579	0.7373	0.0064	−0.0005	0.0023
2000	0.0408 (34)	0.0724 (80)	0.563	0.0563	0.724	0.0067	−0.0017	0.0025
2001	0.039 (34)	0.0717 (80)	0.5441	0.0548	0.7119	0.0070	−0.0030	0.0026
2002	0.0374 (34)	0.0708 (80)	0.5281	0.0533	0.7007	0.0071	−0.0042	0.0028
2003	0.0359 (34)	0.0697 (80)	0.5145	0.0519	0.6905	0.0073	−0.0054	0.0030
2004	0.0345 (34)	0.0685 (80)	0.5029	0.0506	0.681	0.0073	−0.0067	0.0032
2005	0.0332 (34)	0.0673 (80)	0.4928	0.0493	0.6721	0.0074	−0.0080	0.0034
2006	0.0319 (34)	0.066 (80)	0.484	0.0481	0.6639	0.0073	−0.0093	0.0037
2007	0.0308 (34)	0.0647 (80)	0.4764	0.0470	0.6562	0.0073	−0.0107	0.0039
2008	0.0297 (34)	0.0633 (80)	0.4696	0.0458	0.6489	0.0073	−0.0121	0.0042
2009	0.0287 (34)	0.062 (80)	0.4636	0.0448	0.642	0.0072	−0.0136	0.0045
(b) Both case ascertainment and screening effect are considered; APCR with age between 50 and 69.								
1991	0.0743 (69)	0.078 (52)	0.9524	0.0769	0.9657	0.0012	0.0096	0.0007
1992	0.0727 (69)	0.0744 (56)	0.9768	0.074	0.9826	0.0005	0.0082	0.0008
1993	0.0707 (50)	0.0715 (61)	0.9889	0.0713	0.992	0.0002	0.0068	0.0008
1994	0.0676 (50)	0.0695 (69)	0.9733	0.0688	0.9834	0.0006	0.0054	0.0008
1995	0.0648 (50)	0.0679 (69)	0.9551	0.0664	0.9758	0.0009	0.0041	0.0008
1996	0.0623 (50)	0.0663 (69)	0.9396	0.0643	0.9692	0.0012	0.0028	0.0008
1997	0.06 (50)	0.0647 (69)	0.9262	0.0622	0.9634	0.0014	0.0015	0.0009
1998	0.0578 (50)	0.0632 (69)	0.9146	0.0603	0.9583	0.0016	0.0002	0.0009
1999	0.0558 (50)	0.0617 (69)	0.9047	0.0586	0.9538	0.0018	−0.001	0.0009
2000	0.054 (50)	0.0603 (69)	0.896	0.0569	0.9498	0.0019	−0.0023	0.001
2001	0.0523 (50)	0.0589 (69)	0.8886	0.0553	0.9463	0.002	−0.0036	0.001
2002	0.0507 (50)	0.0575 (69)	0.8821	0.0538	0.9433	0.002	−0.0049	0.001
2003	0.0492 (50)	0.0562 (69)	0.8764	0.0523	0.9405	0.0021	−0.0062	0.0011
2004	0.0478 (50)	0.0549 (69)	0.8715	0.051	0.9381	0.0021	−0.0076	0.0011
2005	0.0465 (50)	0.0536 (69)	0.8672	0.0497	0.936	0.0021	−0.009	0.0012
2006	0.0453 (50)	0.0524 (69)	0.8635	0.0485	0.9342	0.0022	−0.0104	0.0013
2007	0.0441 (50)	0.0513 (69)	0.8603	0.0473	0.9325	0.0022	−0.0118	0.0013
2008	0.043 (50)	0.0502 (69)	0.8576	0.0462	0.9311	0.0022	−0.0133	0.0014
2009	0.042 (50)	0.0491 (69)	0.8552	0.0452	0.9298	0.0021	−0.0149	0.0015

For each year of diagnosis, the minimum (MIN), the maximum (MAX), the sample mean (SM), and the sample standard deviation (SSD) of the set of the posterior means of the APCR for each cohort in Taiwan are presented in a row of Table 3(a) and (b). Given a row in Table 3(a) or (b), the age that MIN happened, the age that MAX happened, the ratio of MIN to MAX (RATIO‐1), the ratio of MIN to SM (RATIO‐2) are also included in the corresponding row. SM and SSD are presented for the United States. No ratio was reported for the United States, because they may be negative.

The following remarks are relevant for Taiwan. MINs in Tables 3(a) and (b) are upper bounds of the period effects and both RATIO‐1 and RATIO‐2 are upper bounds of the proportions of the APCRs attributable to period effects in their respective periods, with RATIO‐1 for the cohort showing the largest APCR and RATIO‐2 for the mean APCR. Table 3(a) regards age group 34–80 and Table 3(b) regards age group 50–69; the former is more relevant if period effect reflects only changes in case ascertainment and the latter is more relevant if period effect includes also screening effect. Both Tables 3(a) and (b) report that the lower bounds of the proportion of incidence rate increase attributable to cohort effects increased with calendar year since 1992. It reached a percentage larger than 53.64% in 2009 for the birth cohort 1929 if screening effects are ignored. If screening is considered, it reached a percentage higher than 14.5% in 2009 for the birth cohort 1940.

### Age‐specific rates by year of diagnosis

**Figure 5:**
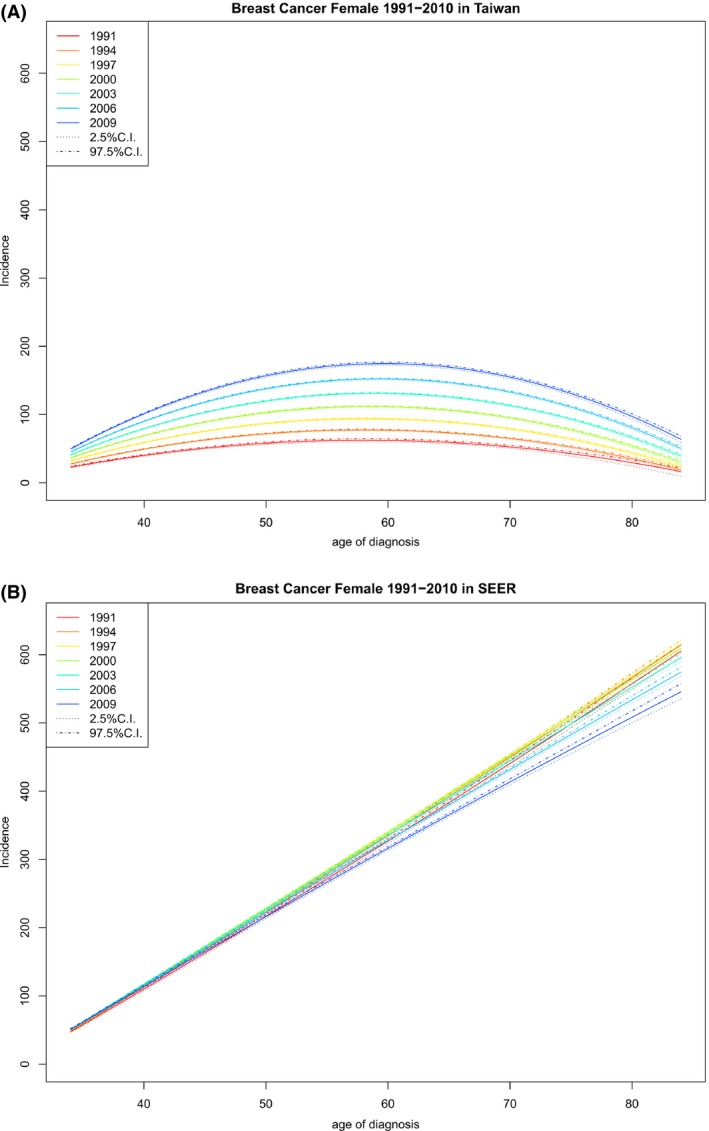
Age‐specific rates by year of diagnosis (rates vs. age at diagnosis, observations within each year of diagnosis are connected) and their 95% credible intervals. (A) Age‐specific rates by year of diagnosis for Taiwan. (B) Age‐specific rates by year of diagnosis for US SEER‐9.

Figure 5 reports age‐specific incidence by year of diagnosis in Taiwan and the US. These figures are in line with the observation that the shape of age‐specific curve is associated with the incidence rate, so called Clememsen's hook 30.

More plots can be found in Figs. S1–S7.

## Discussion

Our findings indicate that (1) for any given birth cohort, the pattern of age‐specific incidence of FIBC in Taiwan and that in the United States were similar (Fig. 1); (2) for a given age at diagnosis, cohort‐specific incidence of FIBC in Taiwan increased monotonically and that in the United States eventually decreased, especially for older cohorts (Fig. 2); (3) given an age at diagnosis, cohort‐specific APCR decreased in both Taiwan and the United States, except for the oldest age groups in Taiwan, although APCRs in Taiwan were always positive and large, compared with those in the United States, which became negative in recent years (Fig. 3); (4) for any specified year of diagnosis after 1995, the APCR in Taiwan decreased considerably with birth year and those in the United States had values near 0 and showed only a slight increase; the proportion of the APCRs attributable to cohort effect were substantial in Taiwan (Fig. 4 and Table 3); and (5) in the period 1991–2010, differences in incidence rates and in their increments between Taiwan and the United States became smaller and were smallest among the youngest cohorts (Fig. 1, Table 1).

All these results were obtained without age–period–cohort model extrapolation and were more informative, compared with Sung et al. (2015, 2016). In particular, comparison of FIBC incidence between Taiwan and the US in terms of cohort‐specific APCR is informative and new; results regarding the proportion of the rate increase that were attributable to cohort versus period effects are also new.

These observations suggest that despite very different period and cohort effects, age exerts its impact on FIBC incidence similarly and independently in Taiwan and in the United States. In particular, for every birth cohort, age‐specific rates did not reach a peak before 75 years of age.

Items 2 and 5 suggest that the FIBC incidence rate in Taiwan has increased rapidly and is likely to continue to do so, which is a public health concern. On the other hand, Items 3 and 4 suggest that this increase has slowed in recent years in view of the decrease in APCR.

Because the data quality of the TCR improved substantially and the completeness of the TCR was approaching optimal, as described in Methods, it is reasonable to assume that period effects did contribute to incidence rate increase and that the APCR due to period effects was decreasing relative to year of diagnosis in the recent past. Thus, in 2009, the proportion of rate increase due to cohort effect is likely to be much higher than 53.64% for the 1929 cohort reported in Table 3(a). This assumption is in line with the decrease in the columns MIN, RATIO‐1, and RATIO‐2 in Table 3a and b since 1993. These results might be considered a first step toward quantifying the proportion of incidence rate increases in Asian countries that are attributable to period effects versus cohort effects.

Although the risk factors contributing to the increasing incidence of FIBC in Asia, including Taiwan, are not fully understood, they are thought to reflect the Westernization of lifestyle, including the consumption of calorie‐dense food, physical inactivity, and obesity 31, 32, 33, in addition to reproduction factors such as early menarche, late childbearing, fewer pregnancies, and use of menopausal hormone therapy, and increased FIBC detection through mammography 34, 35, 36, 37, 38.

Since the 1960s, Taiwan has become increasingly industrialized 39, 40, 41, 42. The Westernization of the citizens’ lifestyle has been suggested as a plausible cause of the rapid increase in FIBC incidence rates in Taiwan 10, 43. These cohort effects and the period effects mentioned above jointly help explain the increase in cohort‐specific rates by age (Fig. 2) and the positivity of the APCRs (Figs. 3 and 4).

With these understandings, we would like to know whether the decrease in cohort‐specific APCR by age, shown in Figure 3, reflect a weakening of the joint period and cohort effects brought about by the lifestyle Westernization in Taiwan. The weakening of the effect due to changes in case ascertainment seems clearly supported by the quality improvement in the TCR, as described above.

Our approach can be used to provide other information about period or cohort effects. For example, considering the MINs for period 2009 in Tables 3(a) and (b), we know that for the 1959 cohort, the proportion of APCR due to case ascertainment effect had an upper bound 0.683 (=0.0287/0.042); hence, that due to screening and cohort effects had a lower bound 0.317 (=1−0.683). But it would be desirable if one could gain better understanding of screening effect by analyzing Taiwan National Screening Program data 44.

As for the cohort effects, it is understandable that the effect of lifestyle Westernization may change with time and it is of interest to describe the effect change over time. In this regard, we would like to point out the possible contributions from various health promotion programs in the past three decades. For example, The John Tung Foundation, founded in 1984, have worked in three fields to promote health: tobacco control, mental health, and nutrition; Formosa Cancer Foundation, founded in 1997, have been systematically promoting healthy life style to reduce cancer incidence, among other things. In addition to various private foundations set up for promoting cancer prevention, Cancer Control Act was promulgated in 2003. A comparison of the Taiwan Nutrition and Health Surveys conducted in 1993–1996 with those in 2005–2008 indicates that some positive dietary and behavioral changes have been observed, including a greater avoidance of products made from animal fats and oils and a concomitant increase in the use of vegetable oil; increased intakes of fruit and vegetables, soy products, fish, whole grains, and nuts and seeds; and reduced intakes of red meat, carbohydrates, and sodium‐containing foods 45. These changes seem to suggest that these health promotion programs are effective, although further studies are needed to establish more specific association between these positive dietary and behavioral changes and decreasing cohort‐specific APCR by age.

The decrease of breast cancer incidence in the United States from 2000 has been considered a consequence of a reduction in the use of menopausal hormone therapy in view of the first report of the Women's Health Initiative, while later studies provided more valuable information 46, 47, 48, 49, 50, 51. Since there was also a reduction in the use of hormone replacement therapy among Taiwanese women aged 40 and older in the period 2001–2004, with the largest drop in 2003 52, 53, more research is warranted to investigate whether this reduction in hormone replacement therapy use contributed to the decline in APCR of FIBC in Taiwan. We note that being based solely on cancer registries and census data, we obtained all the results in this paper without using information about neither screening data nor hormone replacement therapy data.

The results based on SEER data (Figs. 2B and 3B) exemplified that the decrease in cohort‐specific APCR by age preceded the decrease in cohort‐specific incidence rates by age in the United States. Thus, cohort‐specific APCR by age can be used as an early sign for detecting the effects of cancer prevention programs and should be monitored.

Although this paper and recent studies emphasize that age‐specific rates by cohort in Asian are similar to those in the US, this paper also show that, based on Figure 5, age‐specific rates by year of diagnosis was monotonically increasing in the US for every year; those in Taiwan pick between age 55 and 60 and the age that picks increases with year of diagnosis, although slightly. The relation between the shape of age‐specific rates by year of diagnosis and overall incidence rate, exhibited in Taiwan and the US, is in agreement with those observed in Iceland and other countries, which suggest that Clemmesen's hook is due to cohort effect 30.

There are limitations on the results in this paper. Since the purpose of this paper was to compare the age effects and birth cohort effects on FIBC incidence in Taiwan with those in the US white population, our analyses were solely based on cancer registries and census data for the period 1991–2010 and the assumption that FIBC incidence rate varies smoothly on the age range and period range under study. When interpreting the findings, we mentioned some important events like the reduction in the use of menopausal hormone therapy in both Taiwan and the US and the 2003 promulgation of Cancer Control Act in Taiwan, among other things. In fact, the effects of these events on FIBC incidence deserve close investigation. For the effect of Cancer Control Act in Taiwan, for example, one possible approach is to consider and compare several smoothed Lexis diagrams that cover different ranges of years of diagnosis.

Based on this study, additional research is warranted to study the incidence and APCR trends of FIBC in other Asian countries using smoothed Lexis diagram, which may also be useful for the surveillance of other chronic diseases.

## Conflict of Interest

The authors declare that they have no competing interests.

## Supporting information


**Figure S1.** Cohort‐specific rates by period (rates vs. year of birth, observations within each year of diagnosis are connected) and their 95% credible intervals. (A) Cohort‐specific rates by period for Taiwan. (B) Cohort‐specific rates by period for US SEER‐9.
**Figure S2.** Period‐specific rates by cohort (rates vs. year of diagnosis, observations within each birth cohort are connected) and their 95% credible intervals. (A) Period‐specific rates by cohort for Taiwan. (B) Period‐specific rates by cohort for US SEER‐9.
**Figure S3.** Period‐specific rates by age at diagnosis (rates vs. year of diagnosis, observations within same age at diagnosis are connected) and their 95% credible intervals. (A) Period‐specific rates by age at diagnosis for Taiwan. (B) Period‐specific rates by age at diagnosis for US SEER‐9.
**Figure S4.** Age‐specific APCR by year of birth (APCR vs. age at diagnosis, observations within each birth cohort are connected) and their 95% credible intervals. (A) Age‐specific APCR by year of birth for Taiwan. (B) Age‐specific APCR by year of birth for US SEER‐9.
**Figure S5.** Age‐specific APCR by period (rates vs. age at diagnosis, observations within each year of diagnosis are connected) and their 95% credible intervals. (A) Age‐specific APCR by period for Taiwan. (B) Age‐specific APCR by period for US SEER‐9.
**Figure S6**. Cohort‐specific APCR by period (APCR vs. year of birth, observations within each year of diagnosis are connected) and their 95% credible intervals. (A) Cohort‐specific APCR by period for Taiwan. (B) Cohort‐specific APCR by period for US SEER‐9.
**Figure S7.** Period‐specific APRC by cohort (APCR vs. year of diagnosis, observations within each birth cohort are connected) and their 95% credible intervals. (A) Period‐specific APCR by cohort for Taiwan. (B) Period‐specific APCR by cohort for US SEER‐9.

**Data S1.** Methods.
