# Supplementary material for: Comparison of annual percentage change in breast cancer incidence rate between Taiwan and the United States—A smoothed Lexis diagram approach
Source: Cancer Med. 2017 May 31;6(7):1762–75. doi: 10.1002/cam4.1102 (PMC5504335; doi:10.1002/cam4.1102)
Supplement: Supplementary file 1 — Figure S1. Cohort‐specific rates by period (rates vs. year of birth, observations within each year of diagnosis are connected) and their 95% credible intervals. (A) Cohort‐specific rates by period for Taiwan. (B) Cohort‐specific rates by period for US SEER‐9. Figure S2. Period‐specific rates by cohort (rates vs. year of diagnosis, observations within each birth cohort are connected) and their 95% credible intervals. (A) Period‐specific rates by cohort for Taiwan. (B) Period‐specific rates by cohort for US SEER‐9. Figure S3. Period‐specific rates by age at diagnosis (rates vs. year of diagnosis, observations within same age at diagnosis are connected) and their 95% credible intervals. (A) Period‐specific rates by age at diagnosis for Taiwan. (B) Period‐specific rates by age at diagnosis for US SEER‐9. Figure S4. Age‐specific APCR by year of birth (APCR vs. age at diagnosis, observations within each birth cohort are connected) and their 95% credible intervals. (A) Age‐specific APCR by year of birth for Taiwan. (B) Age‐specific APCR by year of birth for US SEER‐9. Figure S5. Age‐specific APCR by period (rates vs. age at diagnosis, observations within each year of diagnosis are connected) and their 95% credible intervals. (A) Age‐specific APCR by period for Taiwan. (B) Age‐specific APCR by period for US SEER‐9. Figure S6. Cohort‐specific APCR by period (APCR vs. year of birth, observations within each year of diagnosis are connected) and their 95% credible intervals. (A) Cohort‐specific APCR by period for Taiwan. (B) Cohort‐specific APCR by period for US SEER‐9. Figure S7. Period‐specific APRC by cohort (APCR vs. year of diagnosis, observations within each birth cohort are connected) and their 95% credible intervals. (A) Period‐specific APCR by cohort for Taiwan. (B) Period‐specific APCR by cohort for US SEER‐9. [file CAM4-6-1762-s001.docx]

Supplementary Figures


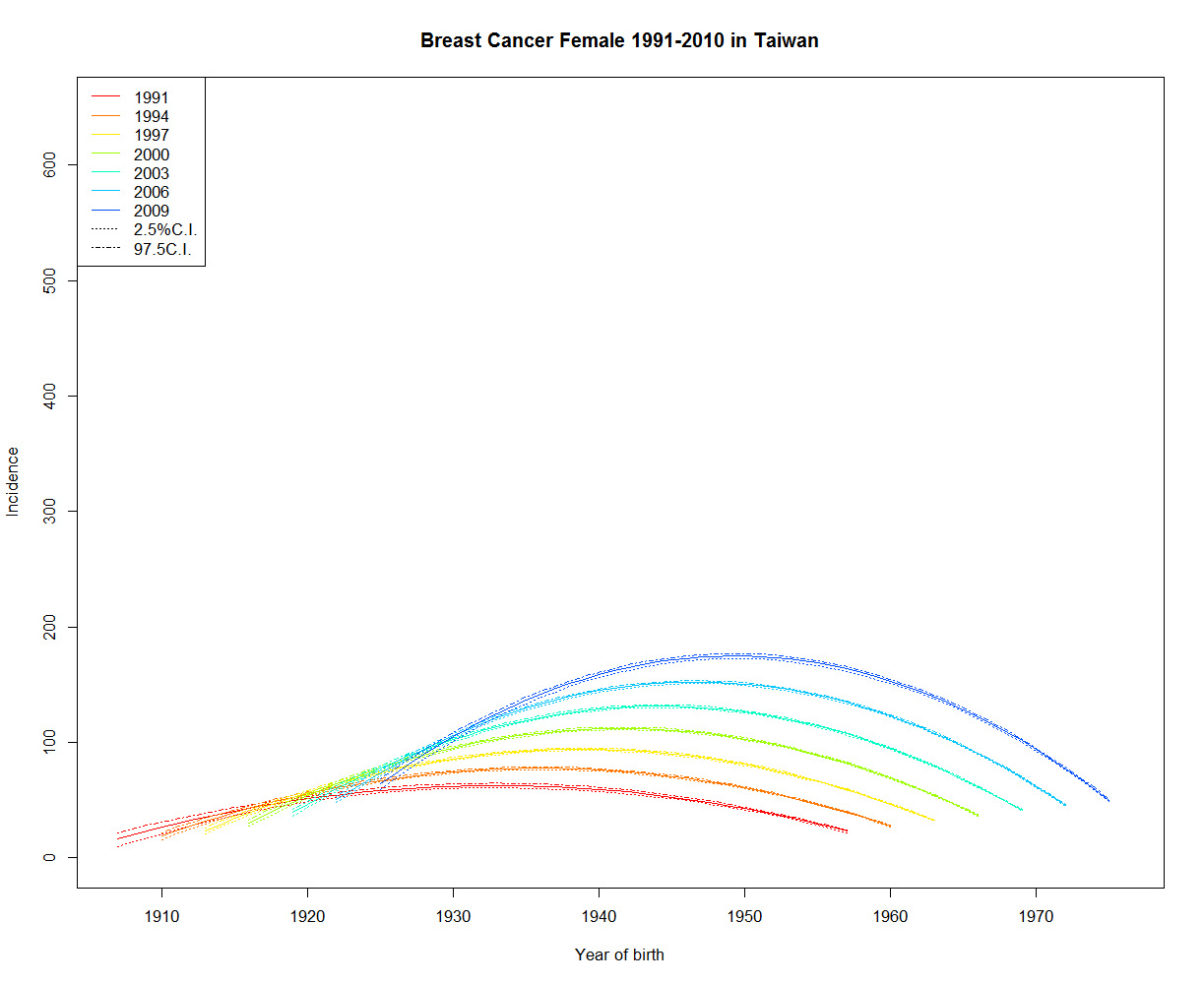

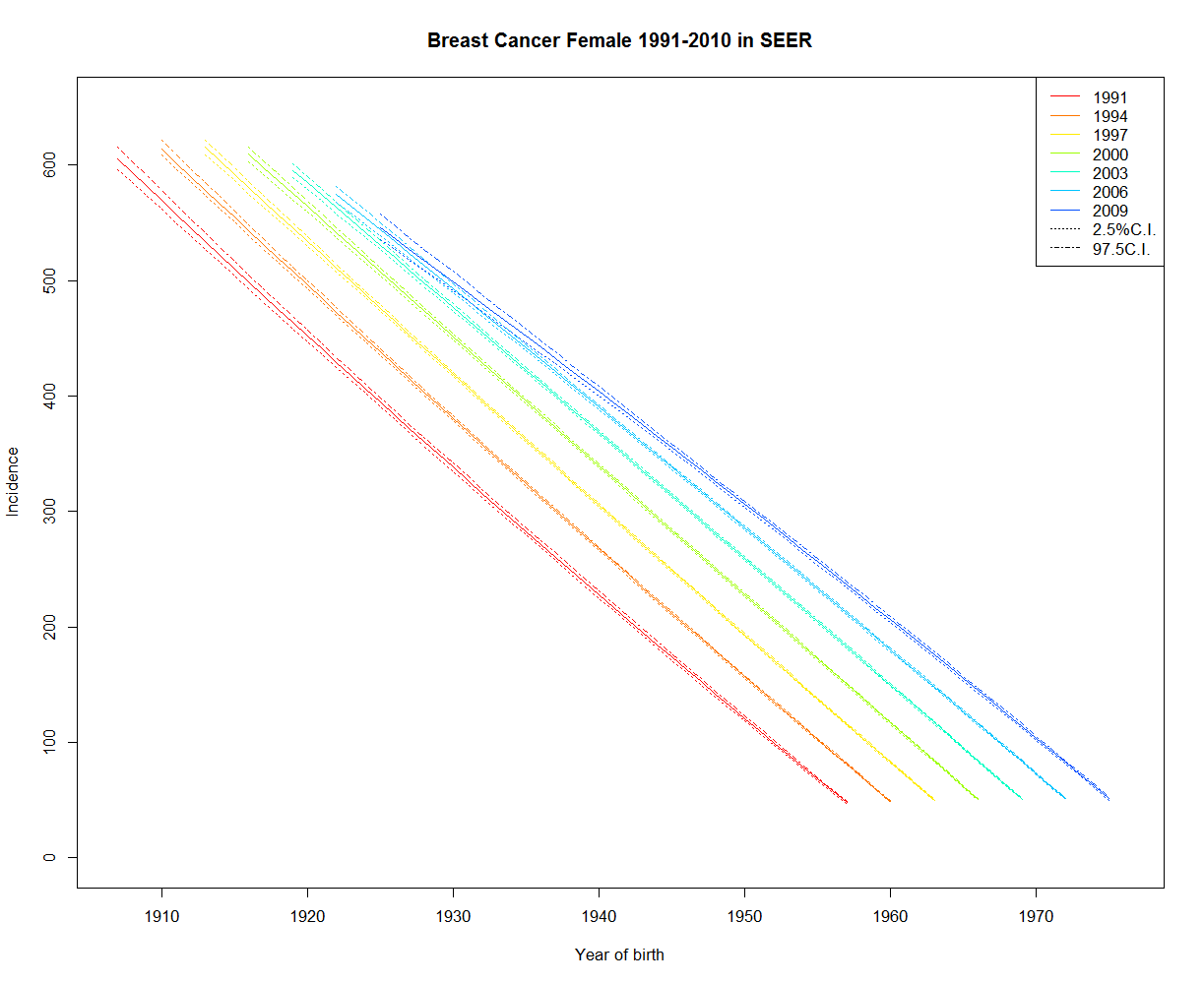


Figure S1A. Cohort-specific rates by period for Taiwan. Figure S1B. Cohort-specific rates by period for US SEER-9.

Figure S1. Cohort-specific rates by period (rates vs. year of birth, observations within each year of diagnosis are connected) and their 95% credible intervals.


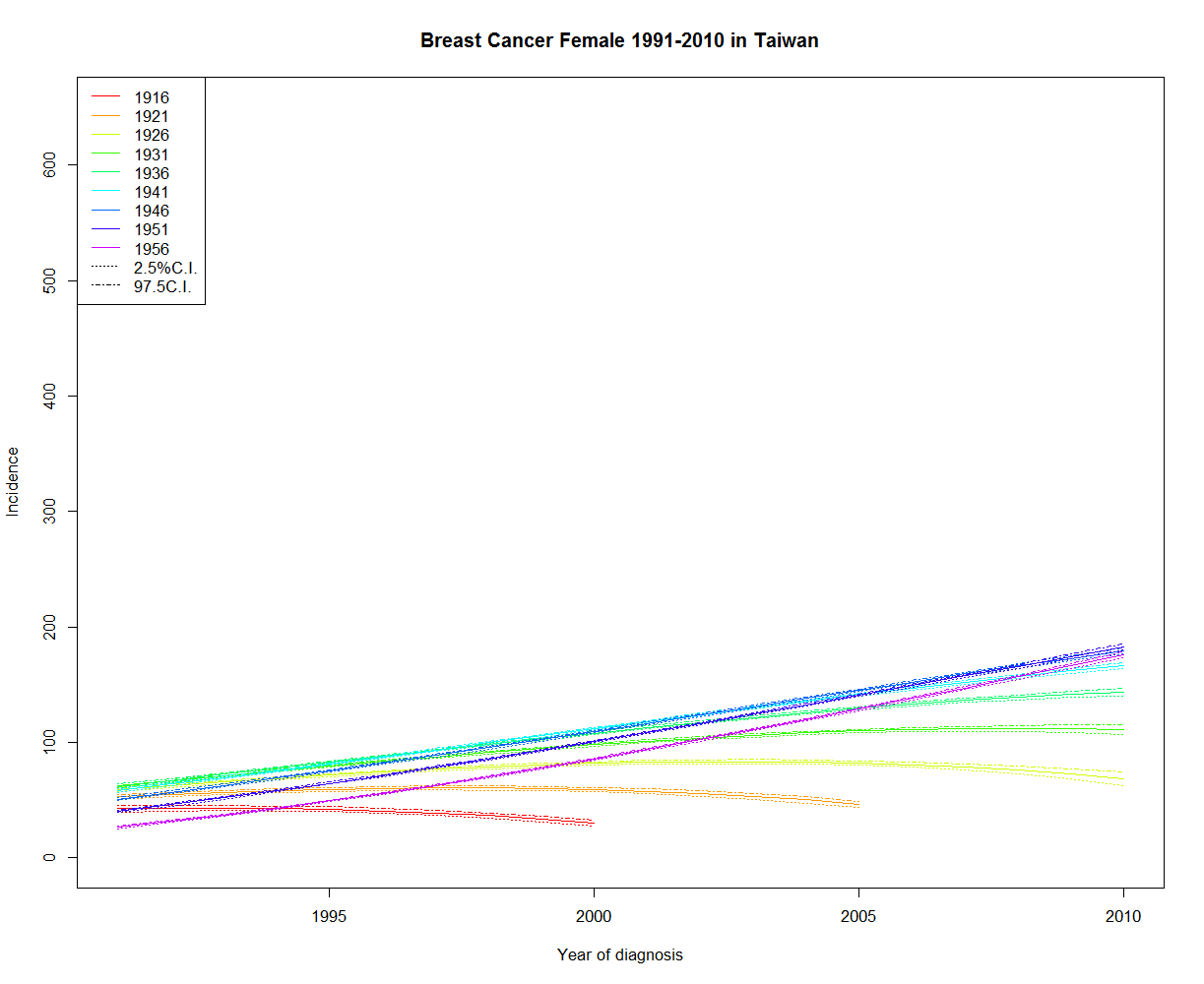

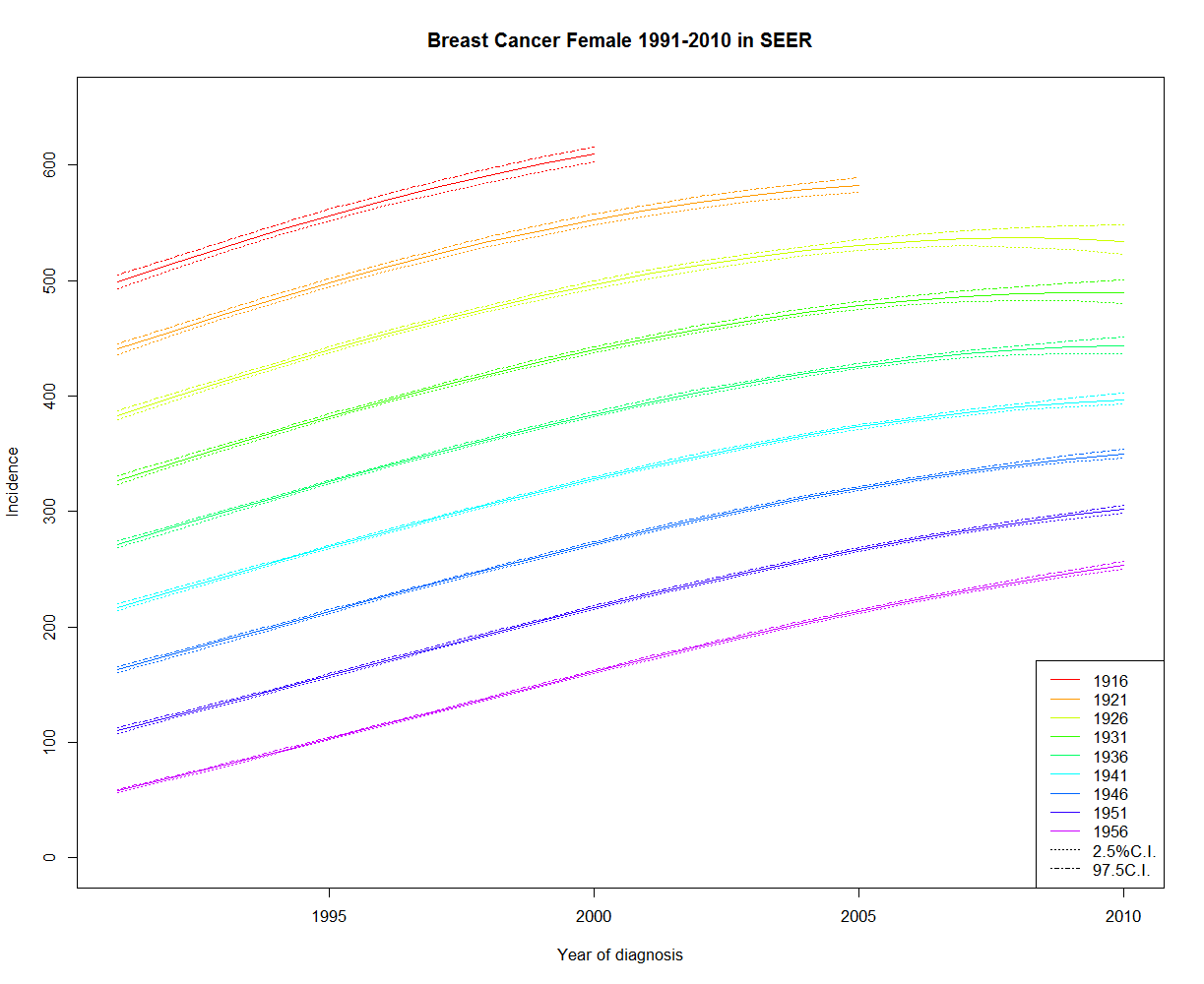


Figure S2A. Period-specific rates by cohort for Taiwan. Figure S2B. Period-specific rates by cohort for US SEER-9.

Figure S2. Period-specific rates by cohort (rates vs. year of diagnosis, observations within each birth-cohort are connected) and their 95% credible intervals.


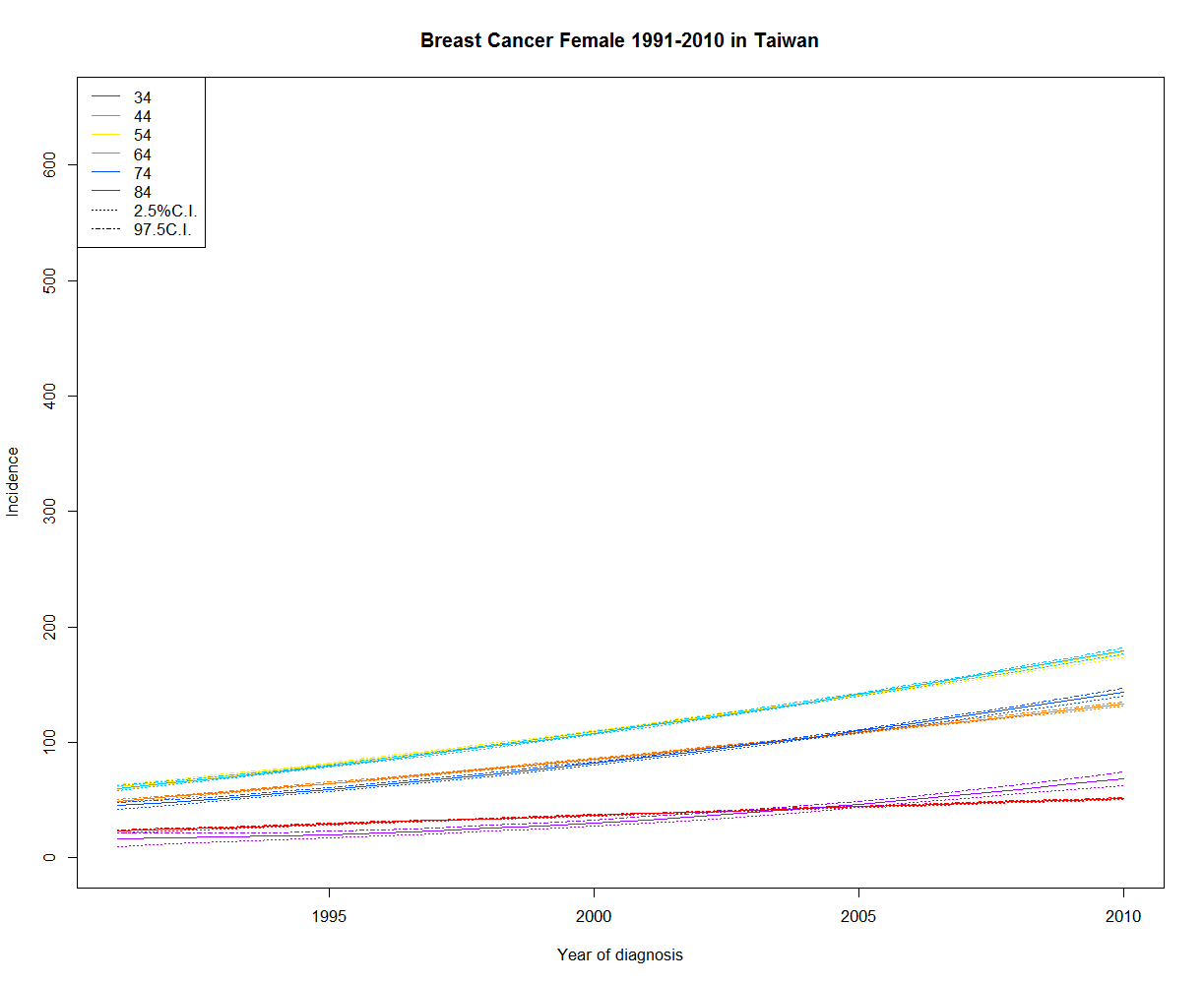

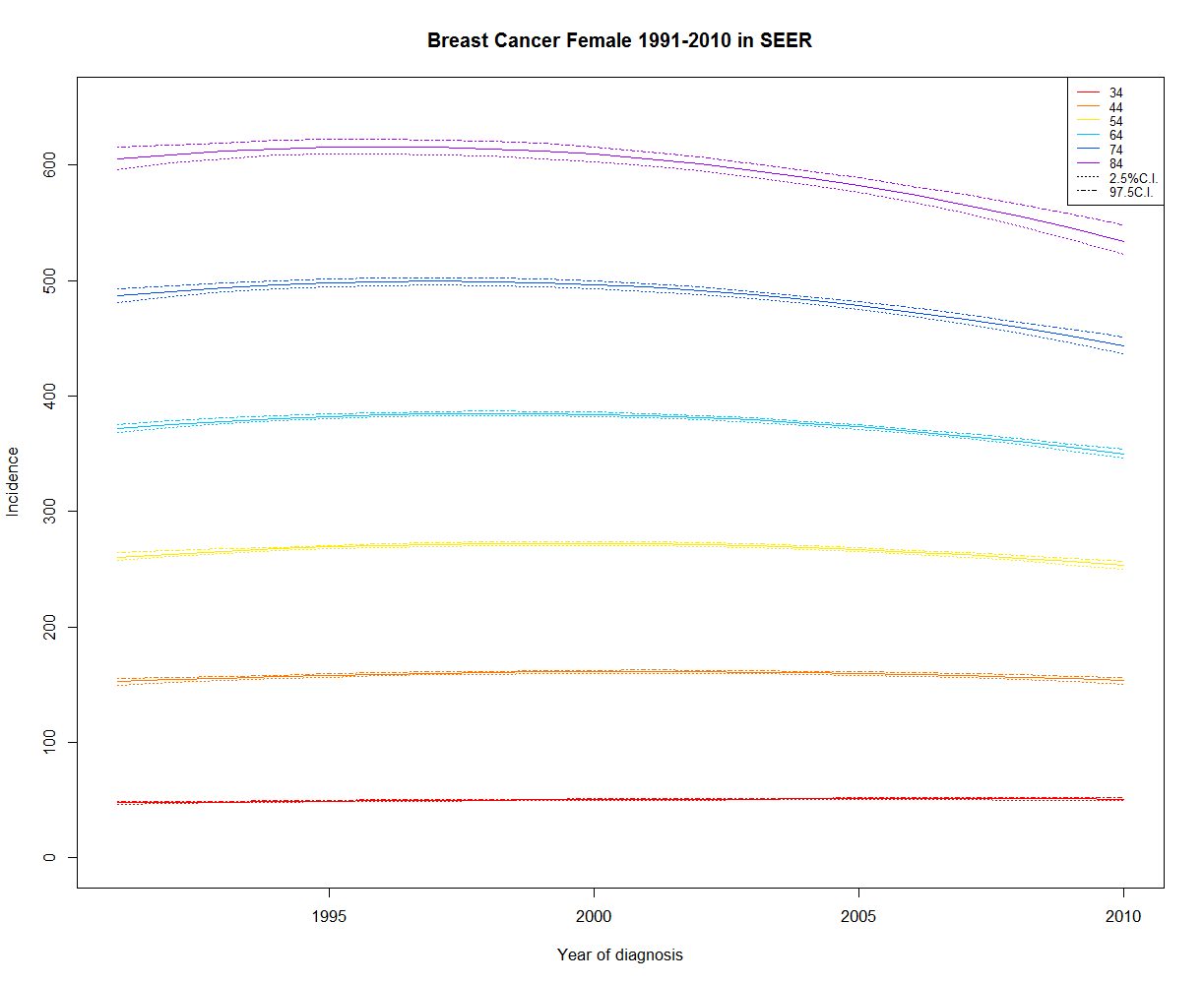


Figure S3A. Period-specific rates by age at diagnosis for Taiwan. Figure S3B. Period-specific rates by age at diagnosis for US SEER-9.

Figure S3. Period-specific rates by age at diagnosis (rates vs. year of diagnosis, observations within same age at diagnosis are connected) and their 95% credible intervals.


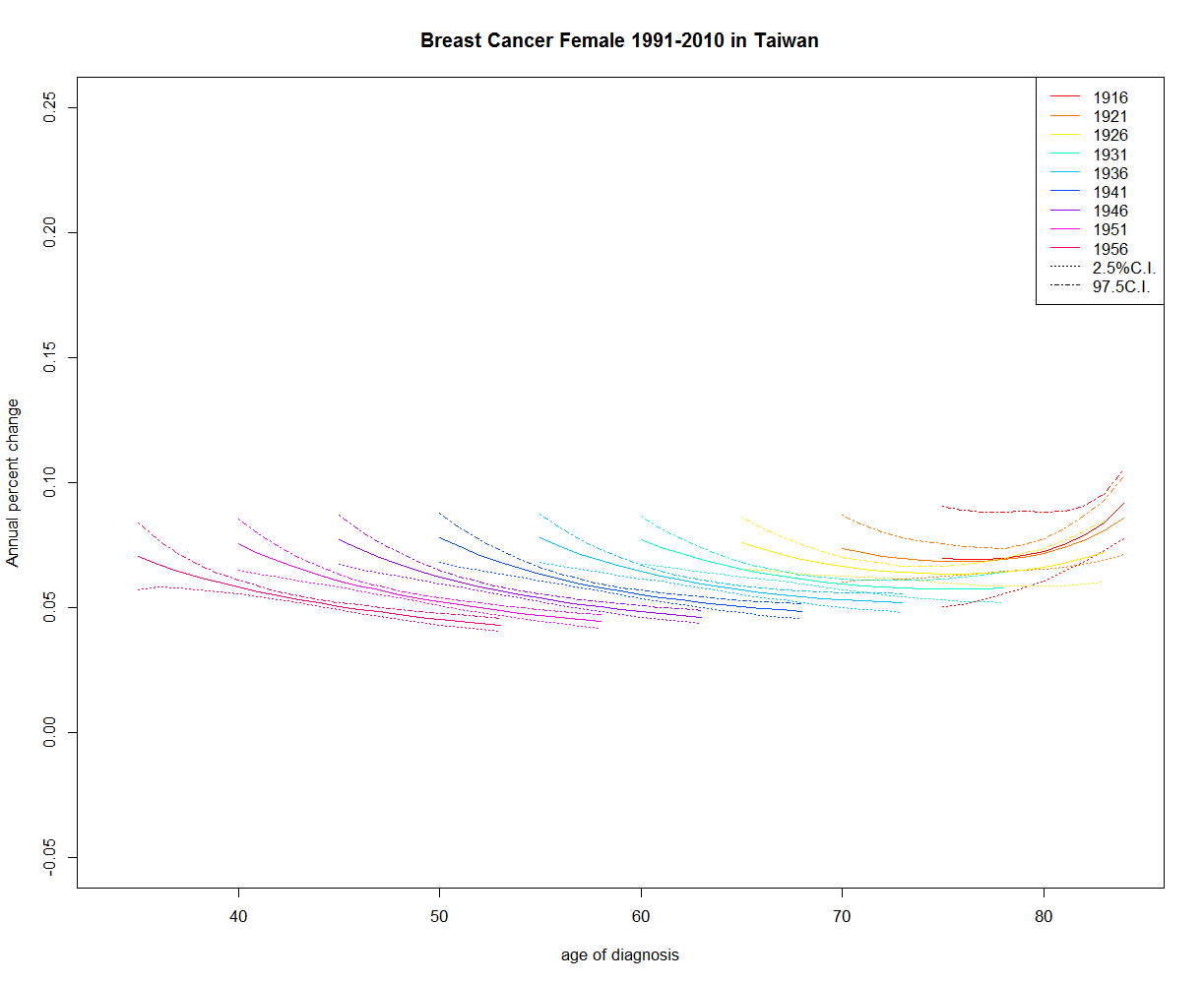

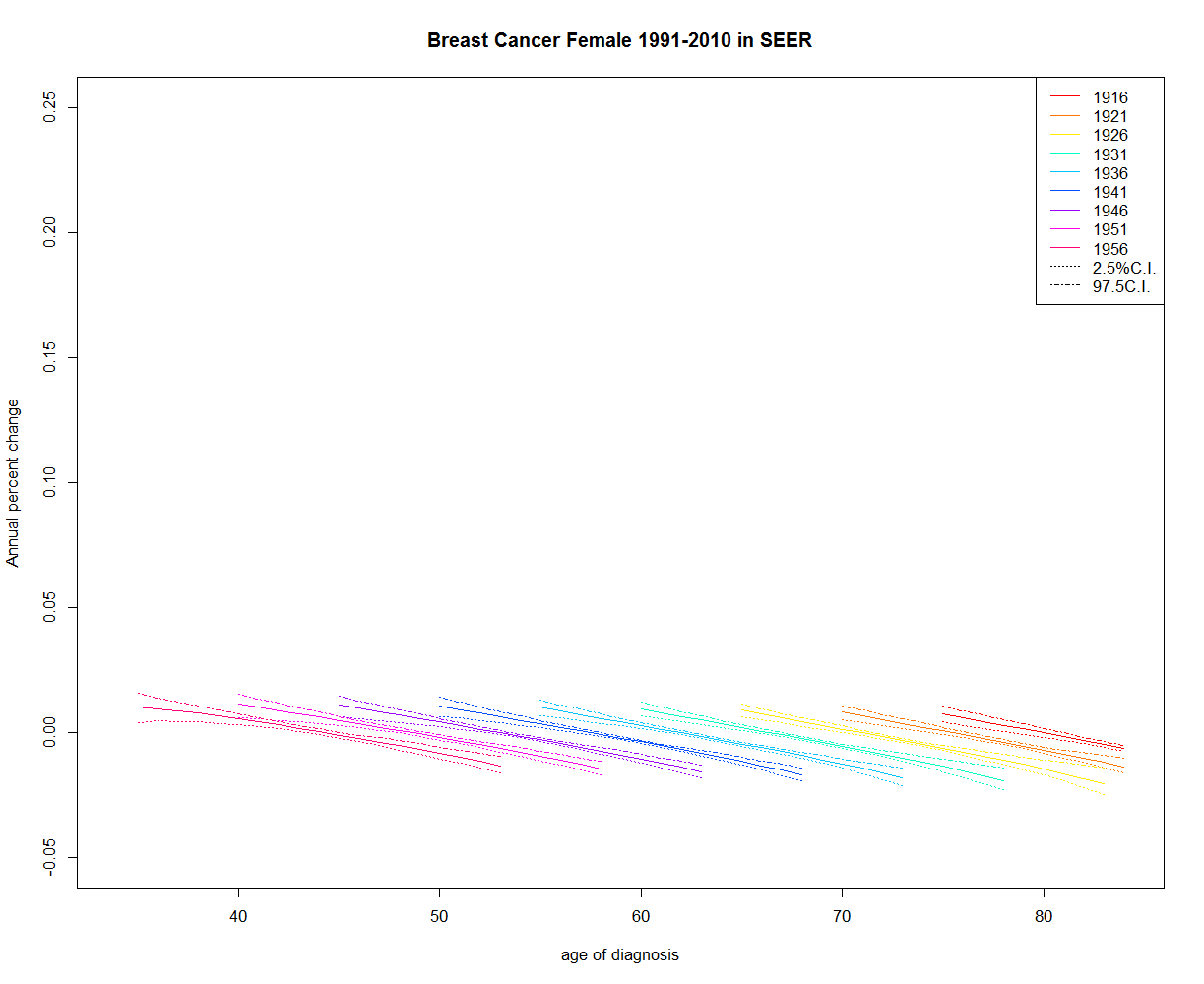


Figure S4A. Age-specific APCR by year of birth for Taiwan. Figure S4B. Age-specific APCR by year of birth for US SEER-9.

Figure S4. Age-specific APCR by year of birth (APCR vs. age at diagnosis, observations within each birth-cohort are connected) and their 95% credible intervals


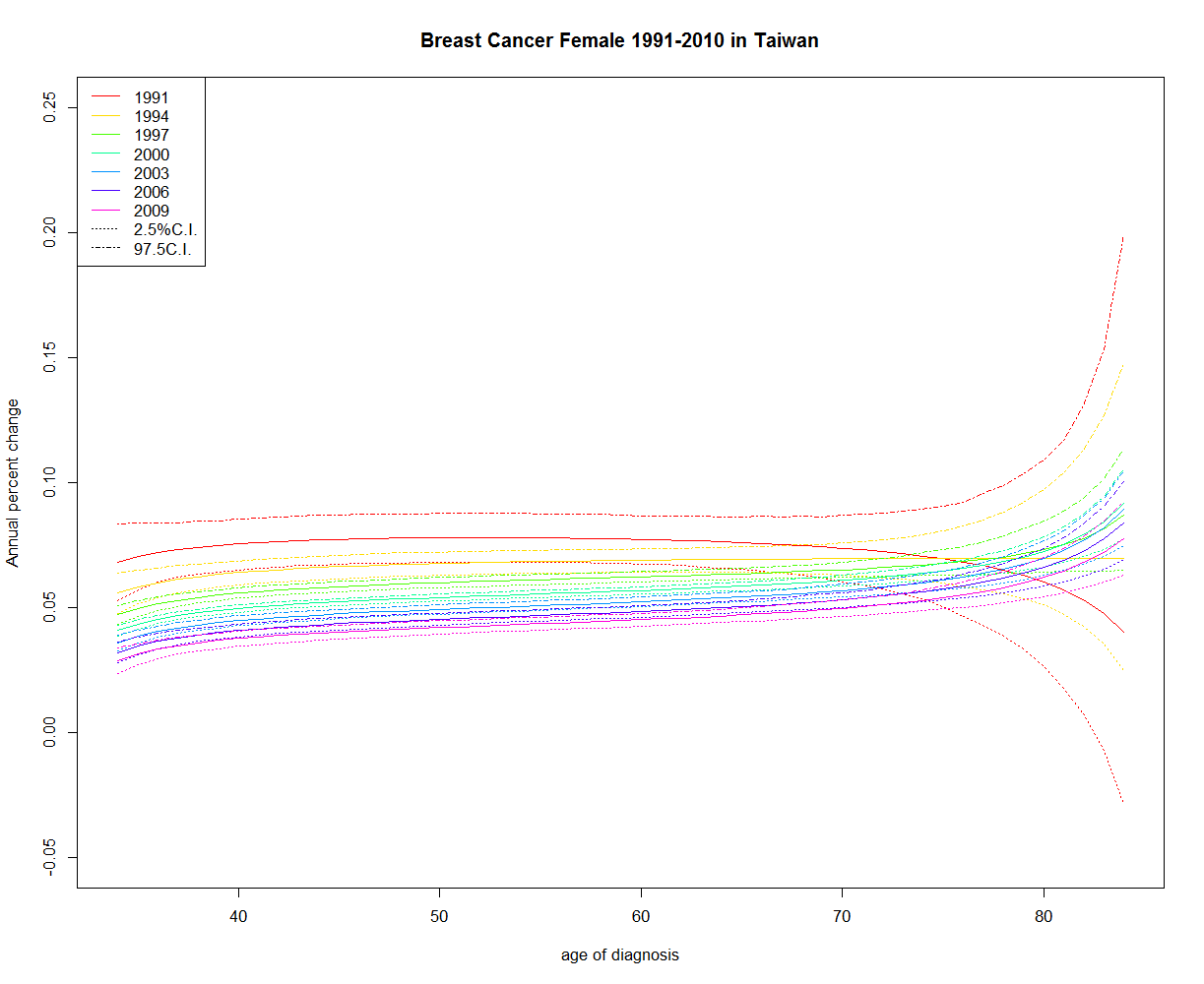

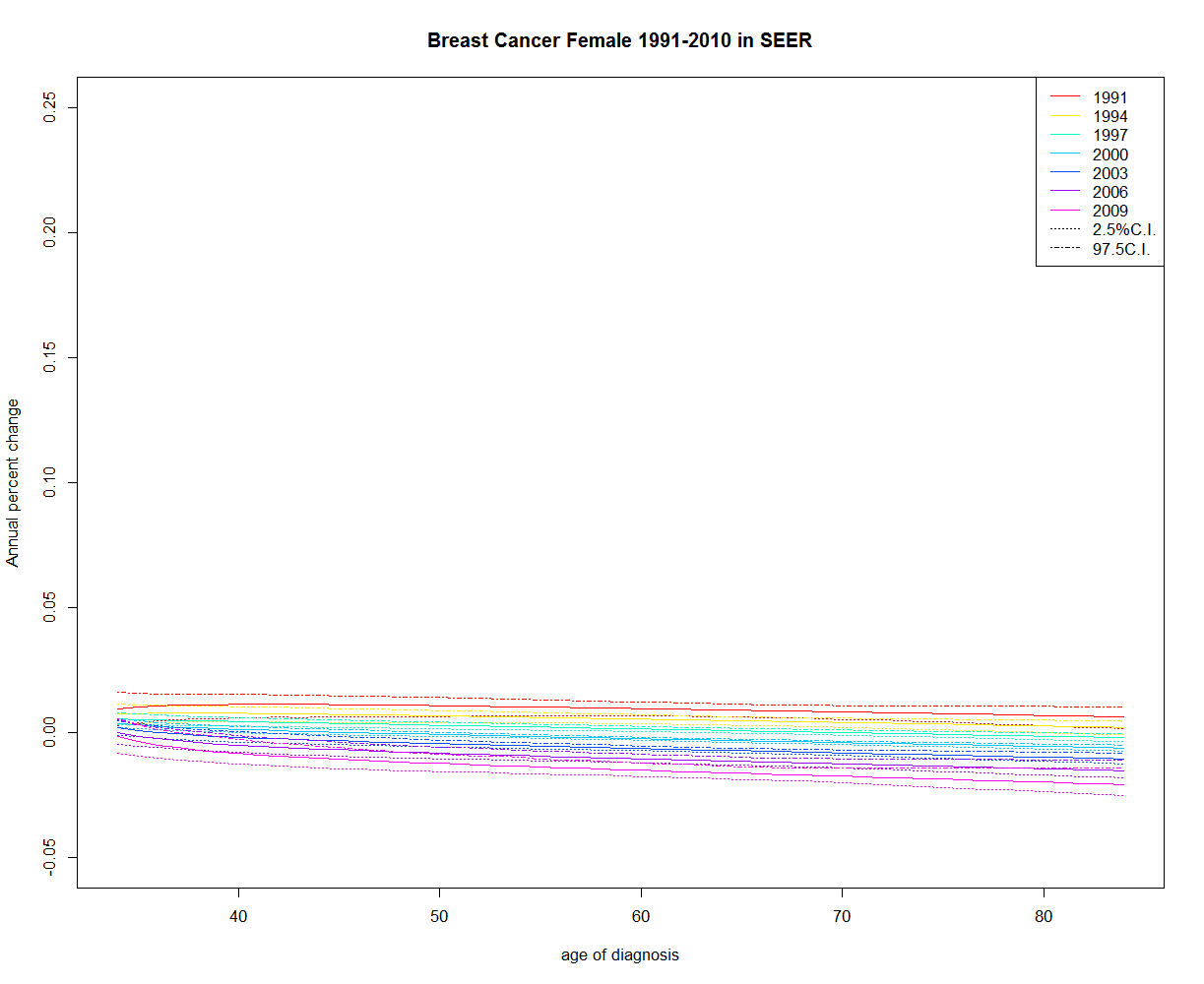


Figure S5A. Age-specific APCR by period for Taiwan. Figure S5B. Age-specific APCR by period for US SEER-9.

Figure S5. Age-specific APCR by period (rates vs. age at diagnosis, observations within each year of diagnosis are connected) and their 95% credible intervals.


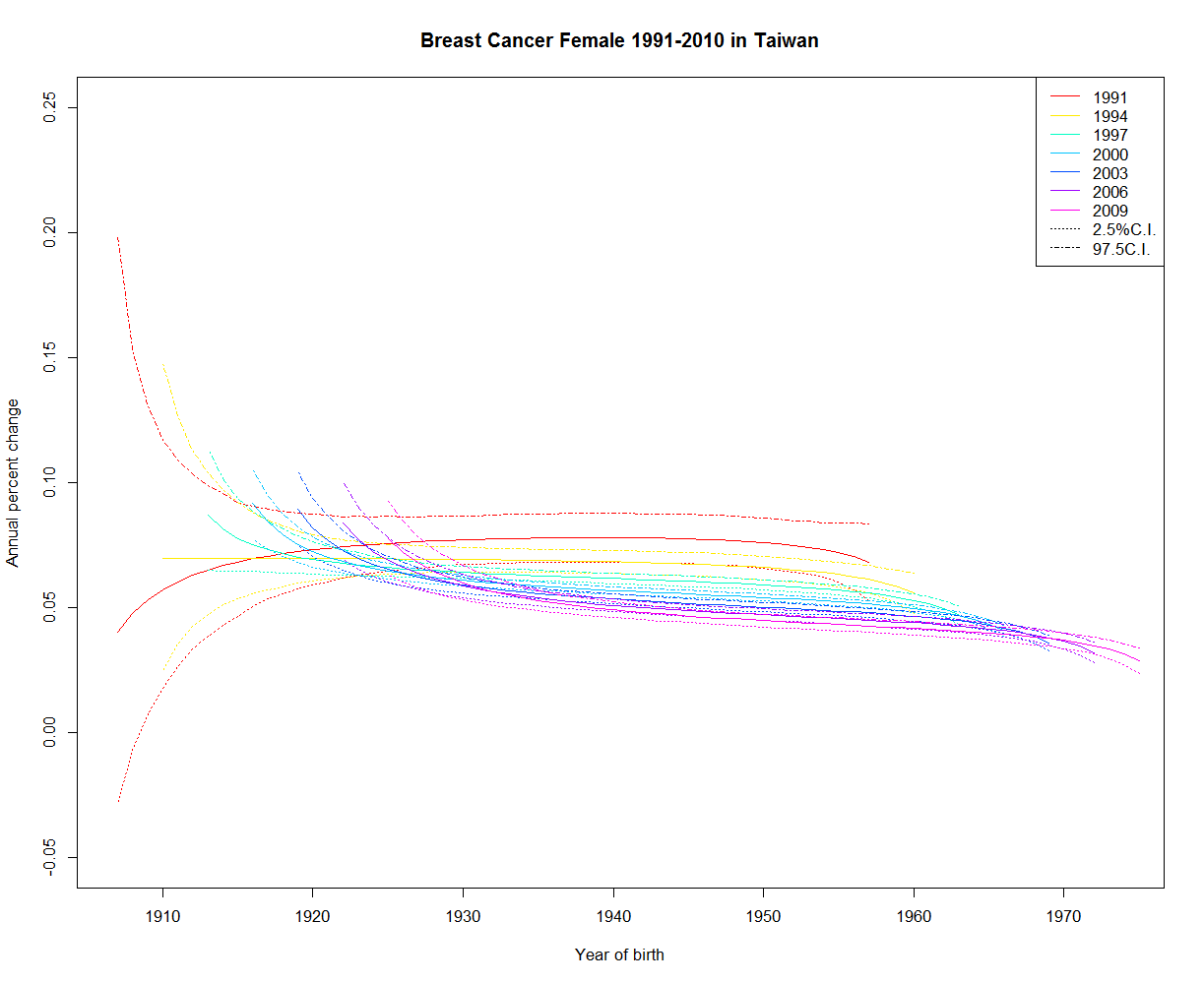

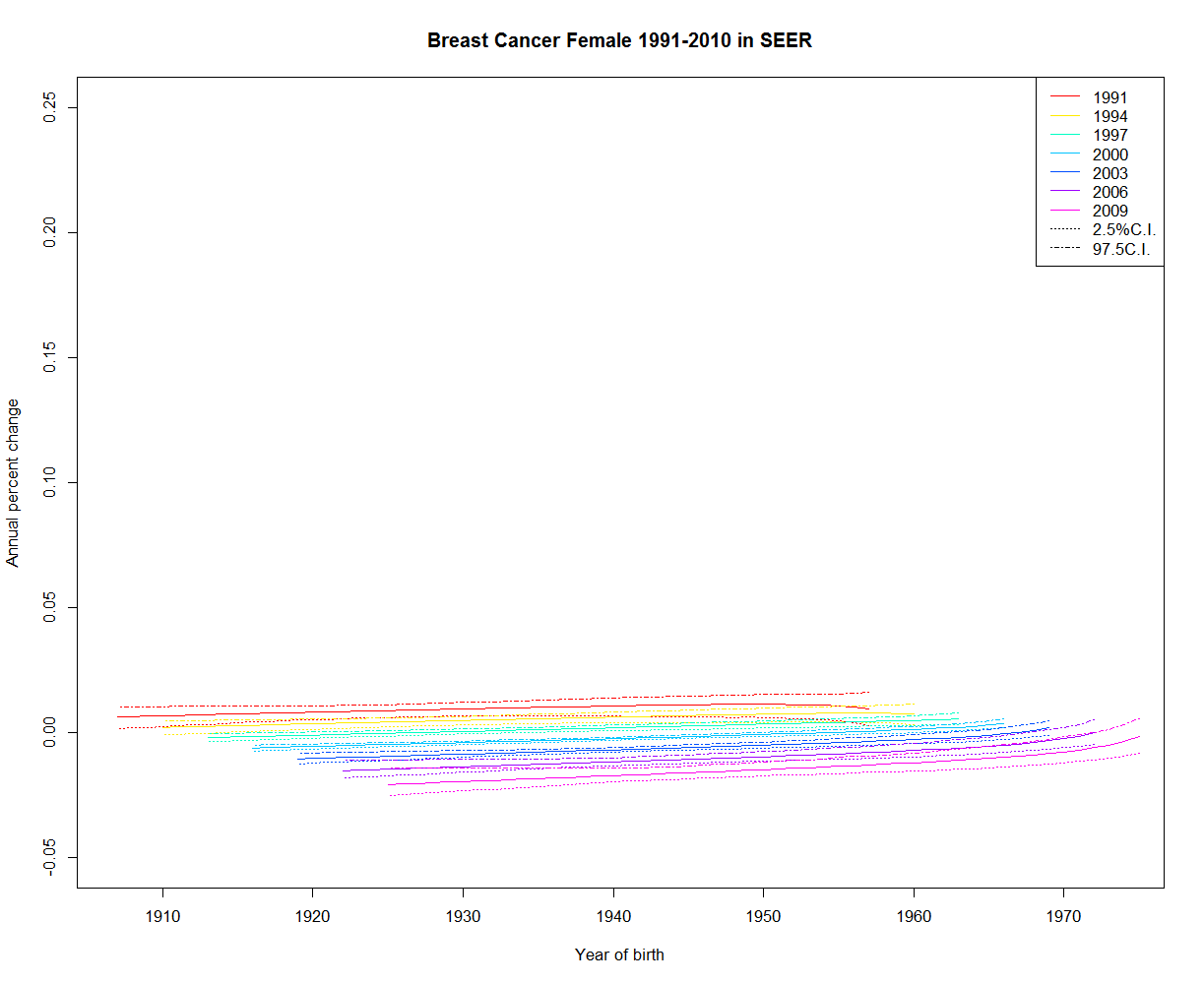


Figure S6A. Cohort-specific APCR by period for Taiwan. Figure S6B. Cohort-specific APCR by period for US SEER-9.

Figure S6. Cohort-specific APCR by period (APCR vs. year of birth, observations within each year of diagnosis are connected) and their 95% credible intervals.


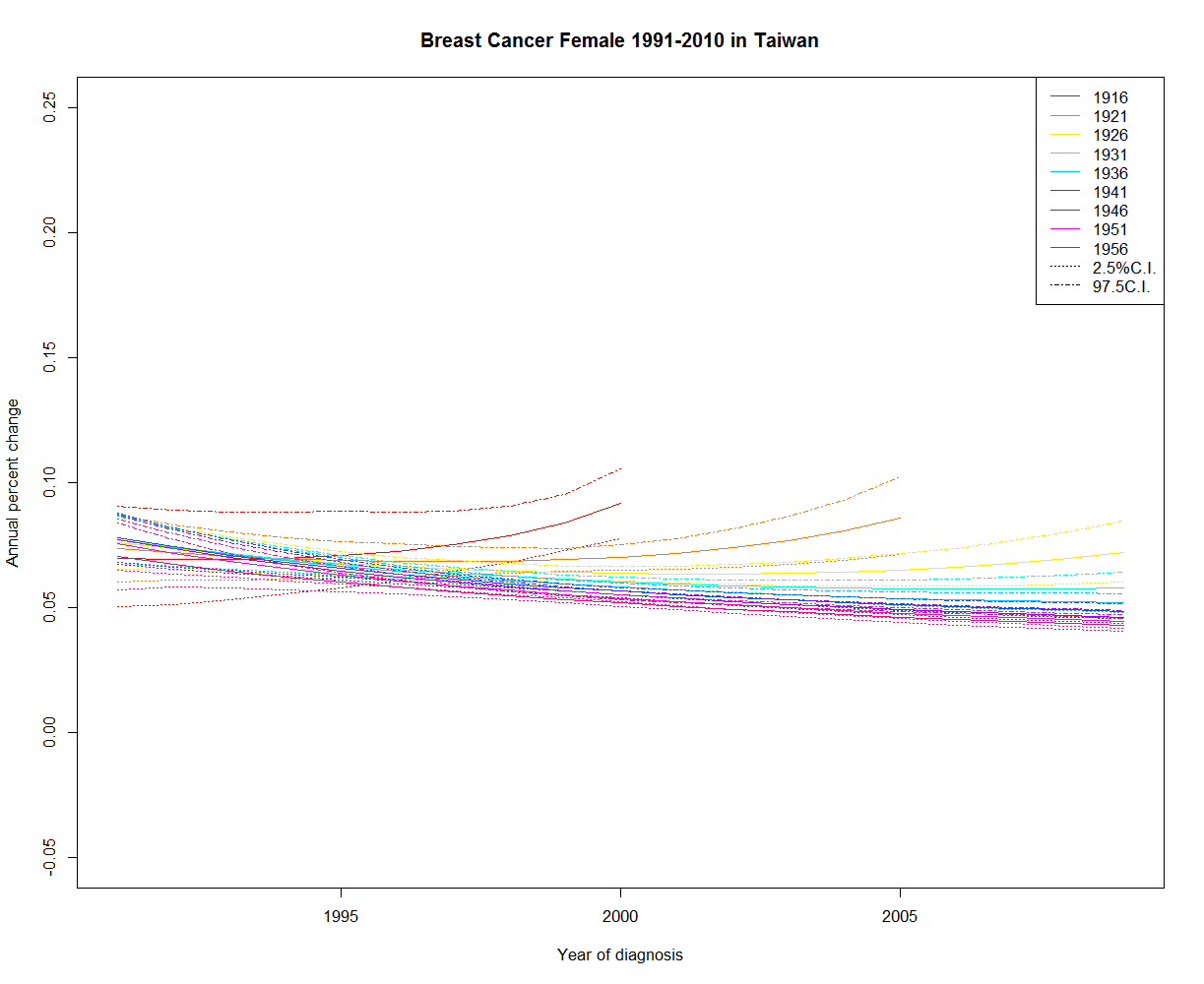

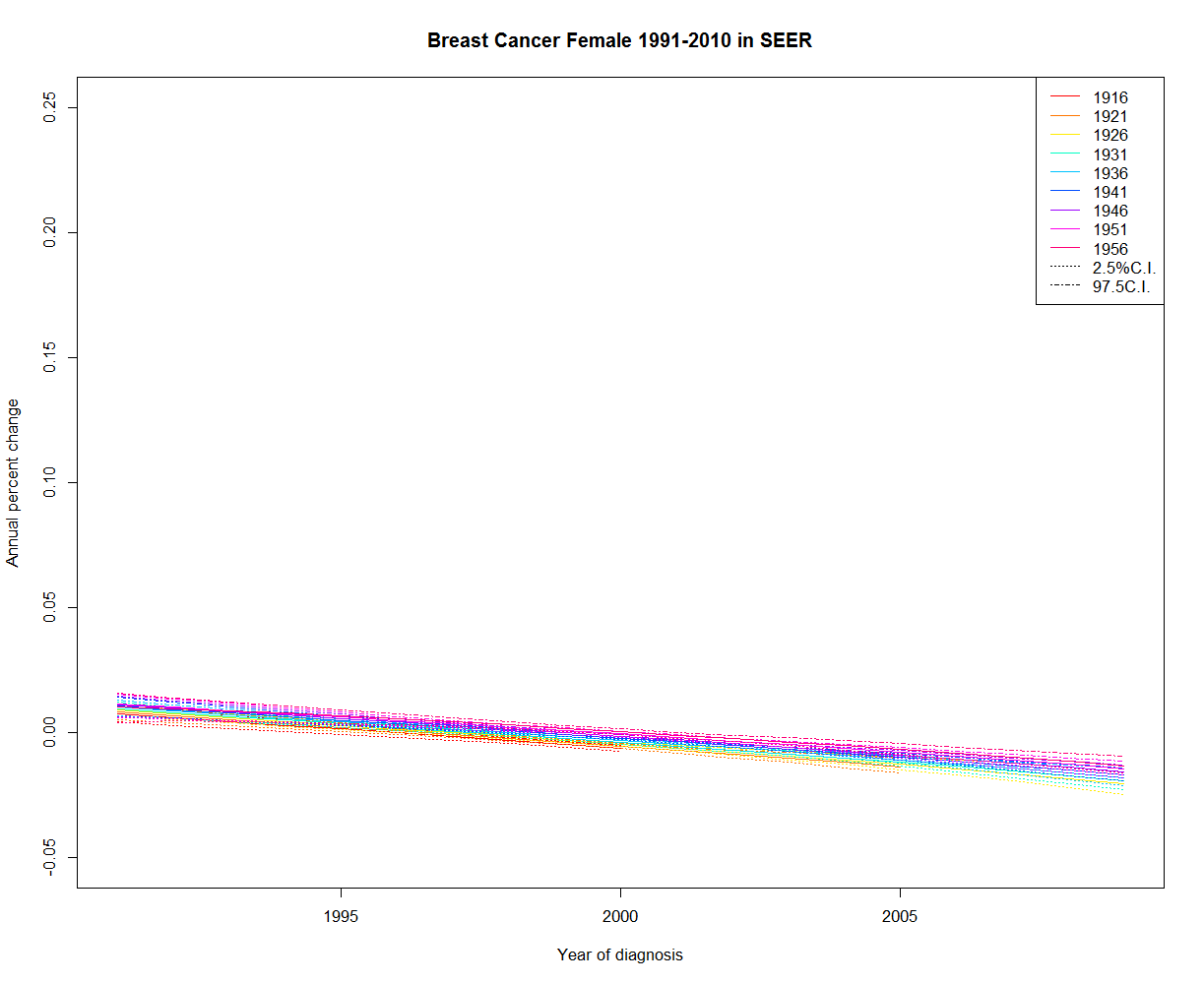


Figure S7A. Period-specific APCR by cohort for Taiwan. Figure S7B. Period-specific APCR by cohort for US SEER-9.

Figure S7. Period-specific APRC by cohort (APCR vs. year of diagnosis, observations within each birth-cohort are connected) and their 95% credible intervals.
