# Supplementary material for: Comparison of annual percentage change in breast cancer incidence rate between Taiwan and the United States—A smoothed Lexis diagram approach
Source: Cancer Med. 2017 May 31;6(7):1762–75. doi: 10.1002/cam4.1102 (PMC5504335; doi:10.1002/cam4.1102)
Supplement: Supplementary file 2 — Data S1. Methods. [file CAM4-6-1762-s002.docx]

**Supplementary Methods**

Statistical analysis

This study used the posterior distribution of the incidence rate function obtained from the Bayesian framework reported by us.(18) Given a population and a specific cancer, the incidence rate function $F(x,y)$ is the probability that an individual will be newly diagnosed with this cancer at age *x* in year *y*. Let $M(x,y)$ denote the number of people in the population not having been diagnosed with the cancer before age *x* and calendar year *y*, and $N(x,y)$ denote the number of people who are newly diagnosed with this cancer at age *x* and in the calendar year *y*. With proper transformation, we assume that both *x* and *y* are equal-spaced fraction numbers in [0,1]. Assume that $N(x,y)$ is Poisson having mean $M(x,y)\text{ }F(x,y)$; thus,

$$P(N(x,y)=z)=\frac{\left\{M(x,y)\text{ }F(x,y)\right\}^{z}}{z!}e^{-\left\{M(x,y)\text{ }F(x,y)\right\}}.$$

We used the MATLAB codes to obtain the posterior distribution of $F$ based on $M(x_{k},y_{k})$ and $N(x_{k},y_{k})$ for $k=1,\ldots,K$; here $(x_{k},y_{k})$ were in $[0,1]\times[0,1]$, $M(x_{k},y_{k})$ and $N(x_{k},y_{k})$ were respectively the one-year tabulated demography and incidence tables. Since cases were diagnosed between 1991 and 2010 and aged between 30 and 84,$K=20\cdot55=1010$.

The posterior distribution of *F* represented by 20,000 samples evenly selected from the 100,000 Markov chain Monte Carlo iterations after the burn-in phase was used to obtain the mean and the 95% credible interval for each point of the classical plot
